# Identification of novel hepatitis B virus therapeutic vaccine candidates derived from polymerase protein

**DOI:** 10.18632/aging.203053

**Published:** 2021-05-20

**Authors:** Juzeng Zheng, Ziqiang Xia, Yilun Xu, Zhanfan Ou, Xianfan Lin, Sisi Jin, Yang Liu, Jinming Wu

**Affiliations:** 1 Department of Gastroenterology, The First Affiliated Hospital of Wenzhou Medical University, Wenzhou 325000, Zhejiang Province, P.R. China; 2 Department of Gastroenterology, Wenzhou People’s Hospital, Wenzhou 325000, Zhejiang Province, P.R. China

**Keywords:** bioinformatics, epitope, hepatitis B virus, polymerase, vaccine

## Abstract

Hepatitis B virus (HBV) infection is a worldwide health problem with high morbidity and mortality rates. The therapeutic vaccine is a promising method of treatment, and HBV polymerase plays a vital role in viral replication. Therefore, a therapeutic vaccine that binds to HBV DNA polymerase may control HBV infection. We predicted and selected epitopes of polymerase using online databases and analysis software. We then performed molecular docking and peptide binding assays to evaluate the binding energies and affinities between polymerase epitopes and the HLA-A0201 molecule. Finally, we induced T cells from the peripheral blood mononuclear cells (PBMCs) of healthy donors using each epitope and quantified the functions of epitope-specific T cells by IFN-γELISPOT assay, T2 cell cytotoxicity assay, HepG2.2.15 cell cytotoxicity assay and HBV gene expression assays. Four epitopes (RVTGGVFLV, GLLGFAAPF, LLDDEAGPL and YMDDVVLGA) had low binding energy and two epitopes (RVTGGVFLV and GLLGFAAPF) had a high binding affinity. The T cells stimulated by two epitopes (GLLGFAAPF and HLYSHPIIL) had a greater ability to induce immune response and suppress HBV. The HBV DNA polymerase epitopes identified in this study are promising targets for designing an epitope-based therapeutic vaccine against HBV.

## INTRODUCTION

Chronic hepatitis B virus (HBV) infection is a worldwide health problem affecting more than 240 million individuals. Most individuals who are infected with HBV are at high risk for developing liver cirrhosis and hepatocellular carcinoma [1]. Approved therapies for chronic hepatitis B (CHB) include two classes of antiviral drugs: nucleos(t)ide analogs and interferon alpha (IFN-α) 2a [2]. Although these two classes of drugs inhibit viral replication, they do not eliminate the virus or its covalently closed circular DNA (cccDNA), the latter of which resides in the nucleus of infected cells [3]. In recent years, scientists have attempted to eliminate HBV and cccDNA using direct-acting antivirals (DAAs) and host-targeting agents (HTAs) [4, 5]. In contrast, therapeutic vaccination work by activating adaptive immunity and is a promising method for both disease prevention and therapy. Such vaccines have effectively induced tumor-specific T cells in patients with pre-cancerous conditions [6, 7].

HBV DNA polymerase (hereafter, “HBV polymerase” or “polymerase”) plays a vital role in viral replication [8]. Maintaining a high level of HBV cccDNA relies on viral replication, so the level of cccDNA in cells will decrease if the polymerase has been inhibited [9]. HBV polymerase interferes with HBV-mediated antagonization of IFN-α, which may explain why IFN-α therapy is not an effective treatment for CHB [10]. Currently the most effective CHB treatment, nucleos(t)ide analogs, suppresses viral replication by inhibiting HBV polymerase, demonstrating that targeting the enzyme is a potentially effective method of CHB treatment.

Chronic HBV infection is characterized by immune tolerance, especially the exhaustion of HBV-specific T cells [11]. Many strategies have been designed to overcome the immune tolerance of HBV [12–15]. A therapeutic vaccine should be able to compensate for immune tolerance. Usually, we can’t detect the HBV polymerase antigen and antibody in the blood, which implies that HBV polymerase rarely comes into contact with the immune system and induces immune tolerance. Therefore, therapeutic vaccines designed based on polymerase peptide epitopes may have the ability to efficiently overcome immune tolerance. Because CD8^+^ T cells can control HBV replication through cytotoxic and non-cytotoxic methods [16], polymerase-specific CD8^+^ T cells may be able to control viral replication without hepatocyte lysis.

In this study, we designed a therapeutic vaccine that targets HBV polymerase with the hope that it would induce an adaptive immune response that could control HBV infection. The human leukocyte antigen (HLA), also named "major histocompatibility complex (MHC), which can present antigenic peptide on the surface of cells. Then, the MHC-peptide compound stimulates T cells through T-cell receptors. Finally, the cellular immune response is activated. Because HLA-A0201 is the most common prevalent MHC-I allele in human [17], we choose HLA-A0201 to predict and identify HBV polymerase epitopes. We used molecular docking databases and analysis software to select polymerase epitopes and calculate the binding energies between the epitopes and the HLA-A0201 molecule. We then performed a peptide binding assay to validate the binding affinities between the polymerase epitopes and HLA-A0201. Next, T cells from human peripheral blood mononuclear cells (PBMCs) were induced by the selected epitopes. The immunogenicity of these epitope-specific T cells was measured by an IFN-γ ELISPOT assay and an *in vitro* T2 cells cytotoxicity assay, and the antiviral abilities of these epitope-specific T cells were determined by an HepG2.2.15 cells cytotoxicity assay and HBV gene expression assay.

## RESULTS

### Epitope prediction and selection

Both the IEDB database and the NetCTLpan 1.1server recommended the same 24 HBV polymerase epitopes (data not shown). Among these 24, the immunogenicity scores of 12 epitopes were positive, and all of the epitopes were nontoxic. Eight epitopes were 100% conserved in the genotype C protein sequence. The results are depicted in Table 1.

**Table 1:** Immunogenicity scores, toxicity and conservation of HBV DNA polymerase epitopes.

**Number**	**Sequence**	**Immunogenicity score**	**^a^Conservation**	**Toxicity**
1	CLFHIVNLI	0.22707	77.78%	Non-Toxin
2	YAAVTHFLL	0.21481	44.44%	Non-Toxin
3	RVTGGVFLV	0.21088	100%	Non-Toxin
4	GLLGFAAPF	0.20787	100%	Non-Toxin
5	HLPDRVHFA	0.20368	100%	Non-Toxin
6	LLDDEAGPL	0.18597	100%	Non-Toxin
7	YVIGSWGTL	0.17599	100%	Non-Toxin
8	YMDDVVLGA	0.11902	100%	Non-Toxin
9	GLSRYVARL	0.0969	88.89%	Non-Toxin
10	GLYRPLLRL	0.05052	88.89%	Non-Toxin
11	WILRGTSFV	0.0468	100%	Non-Toxin
12	HLYSHPIIL	0.04347	100%	Non-Toxin

^a^Amino acid conservation was calculated based on the percentage of epitope sequence that matched the MHC-I genotype C protein sequence.

### Molecular docking and peptide binding assay

**Figure 1:**
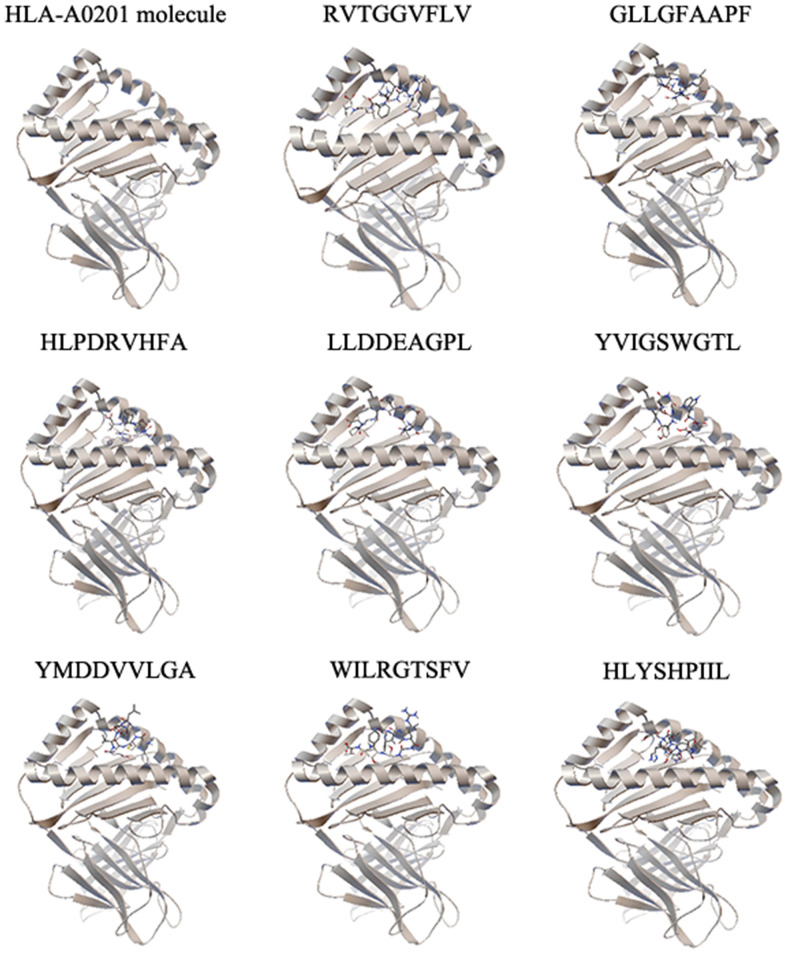
**The molecular docking results of each epitope.** Each epitope binds to the groove of the HLA-A0201 molecule between the α1 and α2 domains, which is shown by secondary structure. One representative of five independent experiments is shown.

To identify the selected epitopes, we performed molecular docking and a peptide binding assay. The binding energies between epitopes and the HLA-A0201 molecule were calculated by molecular docking (Table 2 and Figure 1). We identified the four epitopes (RVTGGVFLV, GLLGFAAPF, LLDDEAGPL and YMDDVVLGA) with the lowest binding energies and selected them from further study. The binding affinities between the selected epitopes and the HLA-A0201 molecule were validated by a peptide binding assay. As shown in Table 3, two epitopes (RVTGGVFLV and GLLGFAAPF) had the highest binding affinities to the HLA-A0201 molecule.

**Table 2:** HBV DNA polymerase epitope binding energies.

**Number**	**Sequence**	**Model 1**	**Model 2**	**Model 3**	**Model 4**	**Model 5**
1	RVTGGVFLV	8.33^a^	13.94	10.67	16.01	8.91
2	GLLGFAAPF	1.58	1.86	0.64	8.7	7.45
3	HLPDRVHFA	22.79	48.45	30.97	131.21	54.74
4	LLDDEAGPL	13.16	6.65	4.88	5.01	20.39
5	YVIGSWGTL	25.66	20.01	17.64	21.9	31.56
6	YMDDVVLGA	9.8	25.87	25.09	14.86	11.3
7	WILRGTSFV	53.88	96.64	50.06	26.57	24.09
8	HLYSHPIIL	59.14	162.05	56.68	69.09	36.03

^a^Binding energies between HBV DNA polymerase epitopes and the HLA-A0201 molecule.

**Table 3:** HBV DNA polymerase epitope fluorescence indices.

**Number**	**sequence**	**FIs^a^**
1	RVTGGVFLV	1.0647
2	GLLGFAAPF	1.3822
3	HLPDRVHFA	0.2920
4	LLDDEAGPL	0.5106
5	YVIGSWGTL	0.8953
6	YMDDVVLGA	0.4242
7	WILRGTSFV	0.4790
8	HLYSHPIIL	0.6279

^a^Fluorescence index (FI) was calculated as described in the Methods section. One representative of 3 independent experiments is shown.

### *In vitro* human IFN-γ ELISPOT assay

**Figure 2:**
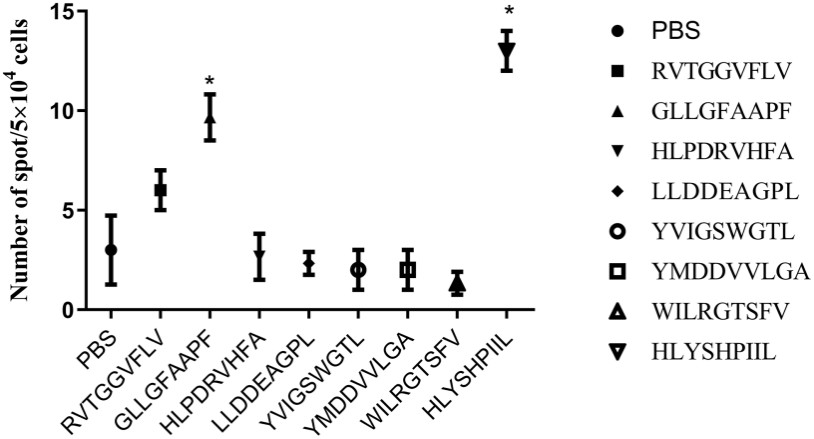
**ELISPOT assay results of each epitope-specific T cell.** We measured the IFN-γ released by T cells obtained from the peripheral blood mononuclear cells (PBMCs) of healthy donors. The number of spots is presented as mean±SD. The mean ± SD from three independent experiments is shown. *Statistically significant compared to the control group.

Because cytotoxic T lymphocytes (CTLs) are known to produce the Th1 cytokine IFN-γ, we quantified epitope-specific T cell responses by measuring IFN-γ secretion with an ELISPOT assay (Figure 2). PBMCs induced by phosphate buffer saline (PBS) were used as a negative control. We concluded that two epitopes (GLLGFAAPF and HLYSHPIIL) generated strong epitope-specific T-cell responses by inducing more IFN-γ secretion than was measured in control (*P*<0.05).

### *In vitro* cytotoxicity assay against T2 cells

**Figure 3:**
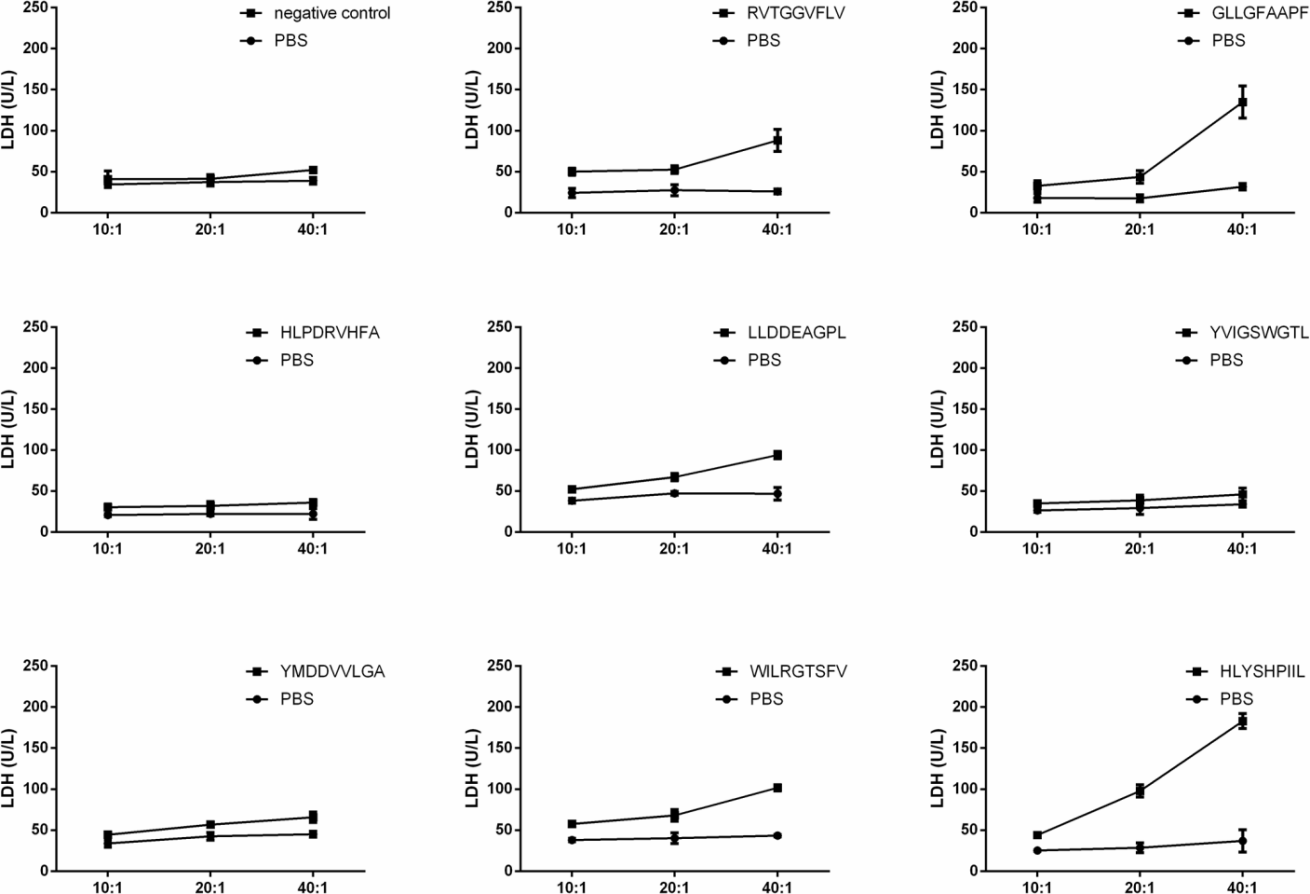
**T2 cell cytotoxicity assay of each epitope-specific T cell type.** T2 cells were pulsed with peptide and lysed by epitope-specific T cells obtained from peripheral blood mononuclear cells (PBMCs). The mean ± SD from 3 independent experiments is shown.

Epitope-specific T cells could lyse antigen-infected T2 cells. We validated the function of epitope-specific T cells by quantifying their cytotoxic activity using a lactate dehydrogenase (LDH) release assay. As shown in Figure 3, the T cells stimulated by the epitopes GLLGFAAPF and HLYSHPIIL exhibited higher cytotoxic activity toward T2 cells compared to T cells stimulated by other epitopes.

### *In vitro* cytotoxicity assay against HepG2.2.15 cells

**Figure 4:**
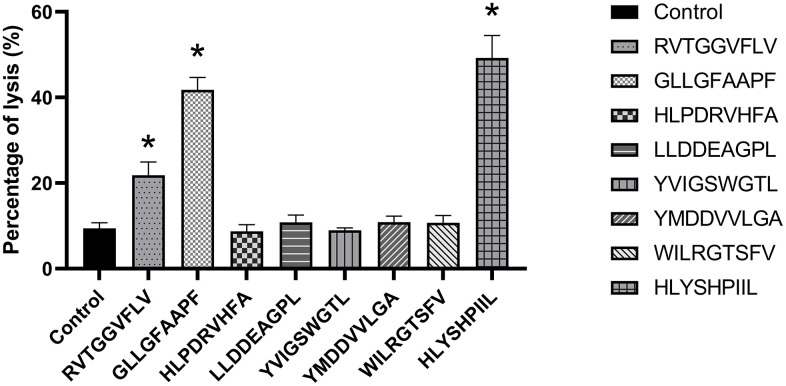
**HepG2.2.15 cell cytotoxicity assay of each epitope-specific T cell type.** HepG2.2.15 cells were lysed by epitope-specific T cells obtained from peripheral blood mononuclear cells (PBMCs). The mean ± SD from 3 independent experiments is shown. *Statistically significant compared to the control group.

In order to determine the antiviral abilities of epitopes, the function of epitope-specific T cells was detected by HepG2.2.15 cell cytotoxicity assay. PBMCs induced by PBS were used as a negative control. As shown in Figure 4, when the ratio of epitope-specific T-cells versus HepG2.2.15 cells was 20:1, the T cells stimulated by the epitopes RVTGGVFLV, GLLGFAAPF and HLYSHPIIL exhibited higher cytotoxic activities against HepG2.2.15 cells compared to control group (*P*<0.05).

### HBV gene expression assays

**Figure 5:**
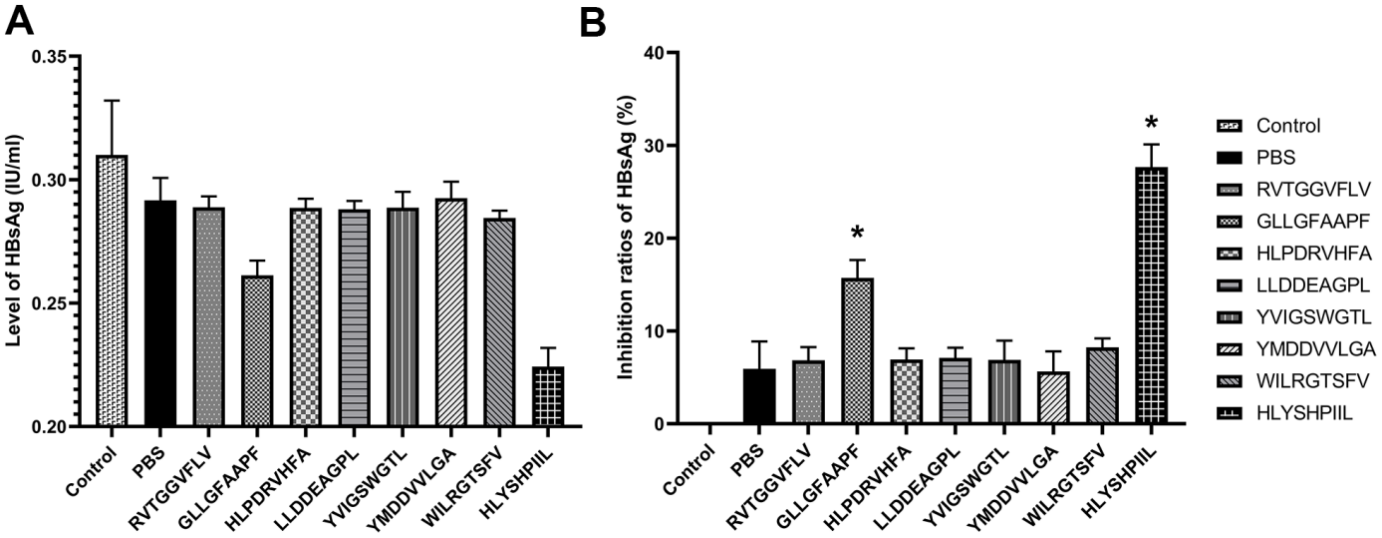
**HBV gene expression assay.** HepG2.2.15 cells were co-cultured with epitope-specific T cells. The HBsAg level of each group was shown in (**A**), and the inhibition ratio of HBsAg was shown in (**B**). The control group was HepG2.2.15 cells cultured without T cells, and the PBS group was HepG2.2.15 cells co-cultured with T cells stimulated by PBS. The mean ± SD from 3 independent experiments is shown. *Statistically significant compared to the PBS group.

**Figure 6:**
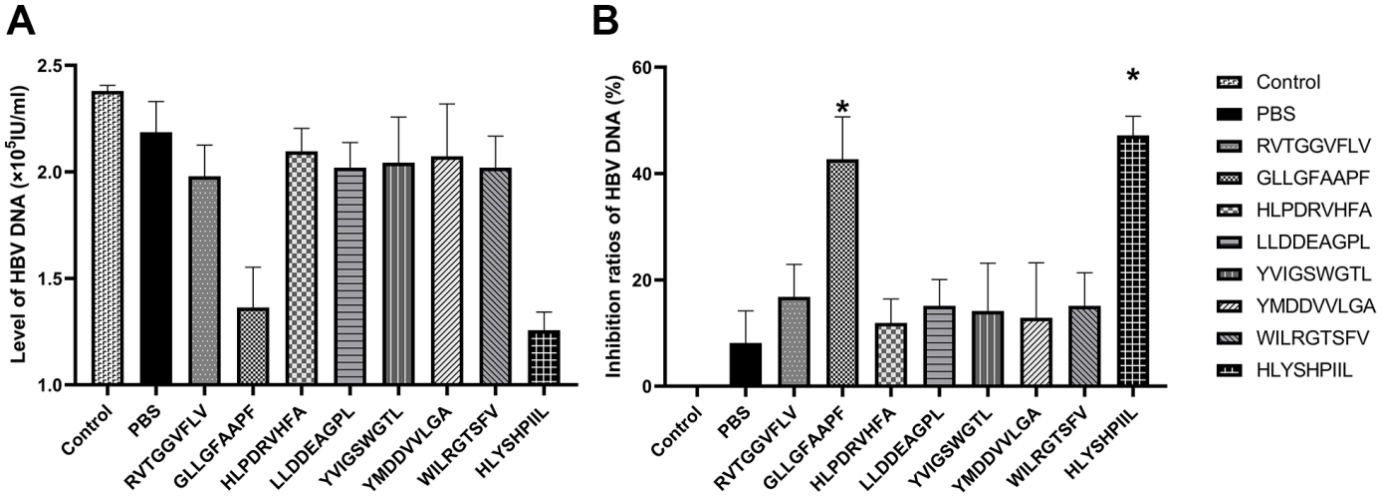
**HBV gene expression assay.** HepG2.2.15 cells were co-cultured with epitope-specific T cells. The HBV-DNA level of each group was shown in (**A**), and the inhibition ratio of HBV-DNA was shown in (**B**). The control group was HepG2.2.15 cells cultured without T cells, and the PBS group was HepG2.2.15 cells co-cultured with T cells stimulated by PBS. The mean ± SD from 3 independent experiments is shown. *Statistically significant compared to the PBS group.

We also determined the antiviral abilities of epitopes using HBV gene expression assays. The antiviral abilities of epitope-specific T cells were validated by calculating the inhibition ratios of HBsAg and HBV-DNA. The control group was HepG2.2.15 cells cultured without T cells, and the PBS group was HepG2.2.15 cells co-cultured with T cells stimulated by PBS. The HBsAg level of each group was shown in Figure 5A, and as shown in Figure 5B, the T cells stimulated by the epitopes GLLGFAAPF and HLYSHPIIL exhibited higher activities to inhibit the expression of HBsAg compared to the PBS group (*P*<0.05). The HBV-DNA level of each group was shown in Figure 6A, and as shown in Figure 6B, the T cells stimulated by the epitopes GLLGFAAPF and HLYSHPIIL had higher abilities to eliminate HBV-DNA compared to the PBS group (*P*<0.05), which was consistent to HBsAg inhibition assay.

## DISCUSSION

In this study, we predicted and identified epitopes of the HBV polymerase protein with the assistance of online databases and analysis software. Eight epitopes conformed to all criteria and were selected for molecular docking and *in vitro* validation. Molecular docking analysis revealed four low binding-energy epitopes (RVTGGVFLV, GLLGFAAPF, LLDDEAGPL and YMDDVVLGA), and the peptide binding assay showed that two epitopes (RVTGGVFLV and GLLGFAAPF) have a high binding affinity for HLA-A0201. An *in vitro* human IFN-γ ELISPOT assay and a T2 cells cytotoxicity assay revealed that two epitopes (GLLGFAAPF and HLYSHPIIL) have a greater ability to induce and stimulate epitope-specific T cells than do the remaining epitopes. A HepG2.2.15 cells cytotoxicity assay showed that three epitopes (RVTGGVFLV, GLLGFAAPF and HLYSHPIIL) had a higher ability to stimulate epitope-specific T cells. Furthermore, in HBV gene expression assays, the results revealed that T cells induced by two epitopes (GLLGFAAPF and HLYSHPIIL) had greater ability to inhibit the expression of HBsAg and HBV-DNA. These epitopes will be further validated by our subsequent studies and might become the basis of polymerase-specific therapeutic vaccines. Although the RVTGGVFLV epitope has a high binding affinity for HLA-A0201 and a high immunogenicity in HepG2.2.15 cells cytotoxicity assay, it had a lesser ability to induce epitope-specific T cells in the IFN-γ ELISPOT, T2 cells cytotoxicity assays and HBV gene expression assays. That the RVTGGVFLV epitope has a high binding affinity to HLA-A0201 implies that it will be presented to the cell surface by the MHC molecule. But a necessary step to induce epitope-specific T cells is that the MHC-epitope compound binds to the T cell receptor and activates it. Although the RVTGGVFLV epitope has a high ability to induce epitope-specific T cells in HepG2.2.15 cells cytotoxicity assay, the ability of RVTGGVFLV epitope is obvious weaker than the other two epitopes (GLLGFAAPF and HLYSHPIIL).

Previously, we selected HBV polymerase epitopes with the help of online databases and analysis tools [18]. We validated these epitopes using molecular docking and a peptide binding assay. The purpose of that study was to select the HBV polymerase epitopes that affect the most HBV-infected individuals in regions of high HBV prevalence. In this study, we selected HBV polymerase epitopes that commonly affect Chinese people and validated these epitopes through *in vitro* experiments.

Chronic HBV infection is a worldwide disease that has high morbidity and mortality rates [3]. Many novel therapeutic methods have been explored in recent years [5], including DAAs and HTAs. Most DAAs are drugs that target and inhibit the steps of viral replication. A drawback to this strategy is that selection and mutation enable viruses to develop drug resistance. Furthermore, drug side effects are inevitable. HTAs, such as innate immune ligands, cellular inhibitors of apoptosis proteins, check-point inhibitors and therapeutic vaccines control viruses indirectly through the immune system [19]. HBV therapeutic vaccines are a promising strategy to inhibit viral replication and eliminate cccDNAs, but the impairment of the immune response and the exhaustion of T cells have limited their utilities [20]. Epitope-based vaccines are designed to induce a precise immune response against antigens and are more efficient than traditional vaccines. Epitopes predicted by bioinformatics tools should be confirmed through *in vitro* and *in vivo* experiments [21]. To surmount these barriers, our present study selected and validated HBV polymerase epitopes that may have the potential to be used in epitope-based vaccines.

Many HBV protective vaccines have been constructed based on the preS, surface and core proteins, but these vaccines have no therapeutic effect on individuals with chronic HBV infection. An HBV polymerase-based vaccine may have the ability to overcome these barriers. In CHB patients, preS, surface and core proteins abound in blood and hepatocytes. They are in constant contact with the immune system, which leads to immune tolerance of these proteins. Thus, vaccines constructed using these proteins are ineffective at eliciting an immune response. In contrast, HBV polymerase mainly hides inside hepatocytes and within HBV, and is rarely in contact with the immune system. Therefore, T cells do not build up a tolerance to the polymerase protein, so vaccines containing polymerase epitopes might not elicit immune tolerance. In addition, preS, surface and core proteins are not required for viral replication, and their elimination by the immune system does not control HBV infection. However, polymerase plays a vital role in HBV replication.

The primary CHB treatment, nucleos(t)ide analogs, targets HBV polymerase and inhibits viral replication, which suggests that HBV infection can be controlled if there is a method that can inhibit polymerase. An HBV polymerase-based vaccine may have the ability to control HBV infection by inducing the immune system to target the HBV polymerase protein. A novel vaccine comprising particulate surface and core antigens, with the saponin-based ISCOMATRIX adjuvant, induces both antigen-specific CD8^+^T cells and antibodies [22]. An antigen-antibody (HBsAg-HBIG) complex therapeutic vaccine candidate with alum as adjuvant (YIC) has been investigated in phase III clinical trial, but the results indicated that overstimulation with YIC decreased the efficacy of the vaccine [23]. In HBV-tolerant mice, a novel particulate vaccine could effectively suppress HBsAg by inducing a humoral immune response. [24]. Moreover, a recent study reported that a yeast-based vaccine candidate expressing X, surface and core antigens could elicit a functional adaptive immune response [25]. Besides, a heat-inactivated, yeast-based T-cell vaccine, GS-4774, could significantly stimulate effective immune response. [26]. Another study tested an HBV DNA vaccine, HB-110, and found that the vaccine exhibited a weaker HBV-specific T-cell induction and HBeAg seroconversion in Korean patients than did the HB-100 vaccine in Caucasian patients [27].

A therapeutic vaccine specifically targeting the HBV DNA polymerase protein has never been reported except in our previous study [18], in which we selected polymerase epitopes *in silico*. TG1050, a non-replicating adenovirus encoding a large fusion protein composed of partial HBV core, polymerase and envelope proteins, has been validated *in vivo*. The results indicated that TG1050 induced both splenic and intrahepatic functional T cells in mice [28]. Because HBV surface and core proteins are not critical in HBV replication, the effect of TG1050 may be attributed to polymerase-specific T cells induced by the polymerase component of this vaccine.

The *in vivo* and *in vitro* experiments were necessary to characterize the epitopes predicted *in silico* [29, 30]. The epitopes in this study were predicted and identified by molecular docking validated with a peptide binding assay and characterized using IFN-γ ELISPOT, T2 cells cytotoxicity, HepG2.2.15 cells cytotoxicity and HBV gene expression assays. Our data indicated that two of these epitopes have the ability to induce and stimulate T cells. In our forthcoming study, epitopes identified in this study will become the basis of novel DNA vaccines [31]. The effectiveness of these DNA vaccines in eliminating the virus will be determined in HBV-transgenic mice.

Our present study has predicted and selected epitopes of the HBV polymerase protein and these epitopes have been validated by *in vitro* experiments. This is a novel and promising strategy for designing therapeutic vaccines. It is possible that a vaccine designed based on the epitopes identified in this study will specially target HBV polymerase and control HBV infection, achieving the same effect of nucleos(t)ide analogues but without drug resistance and the life-long administration of medicine.

## MATERIALS AND METHODS

### Epitope prediction

**Figure 7:**
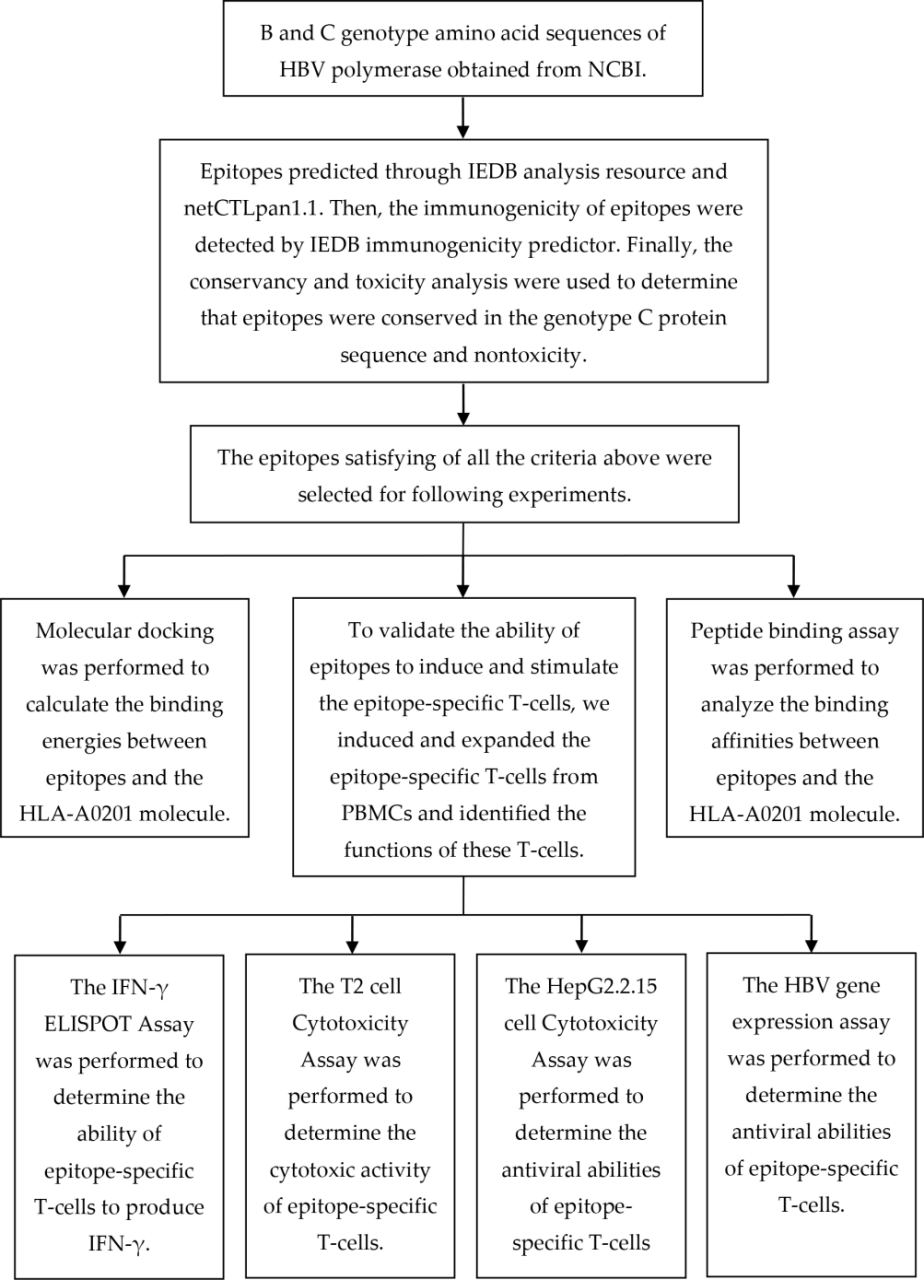
A flow chart illustrating the process of predicting and validating T-cell epitopes.

The epitope prediction was performed based on our previous study [18]. The B genotype protein sequence of HBV polymerase was obtained from the National Center for Biotechnology Information (NCBI) (GenBank accession no. *BAS53332.1*). To predict epitopes that could be presented on the surface of cells, we utilized the MHC-I epitope prediction tools. We selected a nonapeptide within the HLA-A0201 MHC-I allele because most MHC-I epitopes are nonapeptides, and HLA-A0201 is the most prevalent MHC-I allele in human. Other parameters were set to default. The MHC-I epitopes were predicted on 8/7/2016 using the Immune Epitope Database (IEDB) analysis resource consensus tool [32], which combines predictions from NetMHC(3.4) [33, 34], SMM [35] and Comblib [36]. We also used the NetCTLpan 1.1 server to predict peptide MHC-I binding, proteasomal C-terminal cleavage and TAP transport efficiency [37]. We picked the first 30 epitopes (about 3.6%) recommended by each algorithm, and the epitopes repeatedly appeared in first 30 epitopes (predicted by each algorithm) were selected for further analysis. We then used the IEDB immunogenicity predictor [38] to select the epitopes that had a positive immunogenicity value. Finally, we utilized the conservation and toxicity [39] analysis to determine which epitopes were nontoxic and 100% conserved in the genotype C protein sequence (GenBank accession no. *BAQ95565.1*). A flow chart depicting the process of predicting and validating T-cell epitopes is shown in Figure 7.

### Molecular docking

To calculate the binding energies between HBV polymerase epitopes and the HLA-A0201 molecule, we performed molecular docking on the basis of our previous study [18]. Briefly, we downloaded the molecular structure of HLA-A0201 from the RCSB Protein Data Bank and predicted the structures of epitopes using PEP-FOLD2.0 [40, 41]. This yielded five models of structures. The docking analysis was performed by AutoDock4.2 [42]. The number of points in the x-dimension was 60, in the y-dimension was 40 and in the z-dimension was 54. The center grid box was set at x = 0.196, y = 2.472 and z = 20.594. The other parameters were set to default. We calculated the binding energy score and visualized the docking consequence for interaction force analysis. The lower binding energy indicated that epitope was easier bound to HLA-A0201 molecule and harder to be separated.

### Peptide synthesis and cell lines

Epitopes selected by *in situ* methods should be validated by *in vitro* experiments. The epitopes met all the criteria above were synthesized and purified to greater than 99% purity. High-performance liquid chromatography (HPLC) and mass spectrometry were used to analyze the purity and molecular weight of peptides (Shanghai Biotech BioScience and Technology Co, Ltd; Shanghai, China). The TAP-deficient T2 cell line was purchased from the American Type Culture Collection (Manassas, VA, USA).

### Binding affinity of candidate peptides to HLA-A0201

To analyze the binding affinity between epitopes and the HLA-A0201 molecule, we performed a peptide binding assay according to our previous study [18]. Briefly, T2 cells (1×10^6^/well) were incubated with peptides (100 μM) in 24-well plates for 18 hours at 37° C. T2 cells without peptides served as a background control. After being washed with phosphate buffer saline (PBS) three times, cells were stained with FITC-conjugated anti-HLA-A2 mAb BB7.2 and analyzed using a BD AccuriC6 flow cytometer (Becton Dickinson, Mountain View, CA, USA). The mean fluorescence index (MFI) was recorded [43]. We calculated the fluorescence index (FI) as follows: FI = (MFI with individual peptides - background MFI)/(background MFI). Peptides with an FI of greater than 1 were deemed high-affinity epitopes.

### *In vitro* expansion of epitope-specific T cells from PBMCs of healthy donors

To validate the ability of epitopes to induce and stimulate the epitope-specific T cells, we used PBMCs from the blood of healthy donors. The blood was collected after we obtained written informed consent in accordance with procedures approved by the Human Research Ethics Board of the First Affiliated Hospital of Wenzhou Medical University(2017 No. 46) and in accordance with the ethical standards of the Helsinki Declaration. PBMCs were obtained through centrifugation at a Ficoll-Paque density gradient and suspended in RPMI-1640 medium supplemented with 10% fetal bovine serum (FBS), 100 U/ml penicillin and 100 μM streptomycin. PBMCs were stained by FITC-conjugated anti-HLA-A2 mAb and analyzed by flow cytometry. HLA-A0201-positive PBMCs were used for the expansion of epitope-specific T cells. PBMCs were cultured with each peptide (50 μM) and recombinant interleukin 2 (100 U/ml; Sigma, USA). Half of the culture medium was replaced with fresh RPMI-1640 medium every 3 or 4 days. The function of epitope-specific T cells was tested by IFN-γ ELISPOT, T2 cells cytotoxicity, HepG2.2.15 cells cytotoxicity and HBV gene expression assays on the 20th day.

### *In vitro* human IFN-γ ELISPOT assay

In order to measure the ability of epitope-specific T cells to produce IFN-γ, the ELISPOT assay was performed according to the instructions of the kit (Invitrogen, Carlsbad, CA, USA). Epitope-specific T cells(5x10^5^/ml) were added to plates. An automated ELISPOT plate reader was used to quantify IFN-γ release by counting the number of spots present.

### Cytotoxicity assay against T2 cells

T2 cell is (transporter associated with antigen processing, TAP) deficient cell line. When T2 cell is cultured with different epitopes, the HLA-A0201 molecule on surface of T2 cell will bind to different epitopes, the epitope-HLA-A0201 complex can be recognized by epitope-specific T cells and corresponding T2 cell will be lysed. In order to determine the cytotoxic activity of epitope-specific T cells, we measured LDH release based on the instructions of a non-radioactive cytotoxicity assay kit (Promega, USA). Epitope-specific T cells served as effector cells, and T2 cells loaded with or without each peptide served as target cells. The ratios of effector cells versus target cells were set to 10:1, 20:1 and 40:1. The effector cells were co-cultured with target cells (1×10^5^/well) at 37° C for 5 hours, and LDH release was measured.

### Cytotoxicity assay against HepG2.2.15 cell

In order to determine the antiviral abilities of epitope-specific T-cells, we detect the cytotoxicity of epitope-specific T cells against HepG2.2.15 cell. The epitope-specific T-cells served as effector cells, and the HepG2.2.15 cells served as target cells. The ratios of epitope-specific T-cells versus HepG2.2.15 cells were set to 5:1, 10:1, 20:1. The effector cells and target cells were incubated together in 96-well plates at 37° C for 12 hours, and each well was added to total volume of 200 ul. The cytotoxicity activity of epitope-specific T-cells was evaluated based on LDH release using a non-radio-active cytotoxicity assay kit (Promega, US). The percentage of lysis was calculated as formula: (experimental release- target cell spontaneous release- effector cell spontaneous release)/(target cell maximum release- target cell spontaneous release).

### HBV gene expression assay

The antiviral abilities of epitopes were also determined by HBV gene expression assay. The epitope-specific T-cells and HepG2.2.15 cells were co-cultured in 24-well plates at 37° C for 72 hours. The epitope-specific T-cells served as effector cells, and the HepG2.2.15 cells served as target cells. The ratio of effector cells versus target cells was set to 10:1, the density of target cell was 1×10^5^/well. After 72 hours cocultivation, the supernatants were collected to determine the concentrations of HBV surface antigen (HBsAg) and HBV-DNA. The HBsAg concentration was detected by enzyme-linked immunosorbent assay (ELISA) according to the manufacturer’s protocol (Shanghai Siic Kehua Biotech Co., Ltd.). And the HBV-DNA concentration was detected by real-time fluorescence quantitative PCR according to the manufacturer’s protocol (Yaneng Bioscience (Shenzhen) Co., Ltd.). The control group was HepG2.2.15 cells cultured without T cells, and the PBS group was HepG2.2.15 cells co-cultured with T cells stimulated by PBS. The inhibition ratios of HBsAg and HBV-DNA were calculated as the formula: (1- experimental group/ control group)×100%.

### Statistics

All experiments were performed independently 3 times, and the results were reported as mean±SD. We conducted the statistical analysis using a Student's *t*-test. A *P* value <0.05 was considered significant. All statistical analyses were conducted using SPSS 19.0 software.
